# Functional type 1 regulatory T cells develop regardless of *FOXP3* mutations in patients with IPEX syndrome

**DOI:** 10.1002/eji.201040909

**Published:** 2011-01-14

**Authors:** Laura Passerini, Sara Di Nunzio, Silvia Gregori, Eleonora Gambineri, Massimiliano Cecconi, Markus G Seidel, Giantonio Cazzola, Lucia Perroni, Alberto Tommasini, Silvia Vignola, Luisa Guidi, Maria G Roncarolo, Rosa Bacchetta

**Affiliations:** 1 San Raffaele Telethon Institute for Gene Therapy (HSR-TIGET), Division of Regenerative Medicine, Stem Cells and Gene Therapy, San Raffaele Scientific InstituteMilan, Italy; 2 Pediatrics Department, Anna Meyer Children's Hospital, University of FlorenceFlorence, Italy; 3 Human Genetics Laboratories, Ospedale GallieraGenoa, Italy; 4 Stem Cell Transplantation Unit, St. Anna Children's Hospital and Children's Cancer Research InstituteVienna, Austria; 5 Cystic Fibrosis CenterVerona, Italy; 6 IRCCS Burlo Garofalo, Paediatric Immunology LaboratoryTrieste, Italy; 7 Paediatric Gastroenterology, G. Gaslini HospitalGenoa, Italy; 8 OU of Internal Medicine and Gastroenterology, Complesso integrato Columbus, Università Cattolica del Sacro CuoreRoma, Italy; 9 Università Vita-Salute San RaffaeleMilan, Italy

**Keywords:** Forkhead box p3, IL-10, IPEX, Type 1 regulatory T cells

## Abstract

Mutations of forkhead box p3 (*FOXP3*), the master gene for naturally occurring regulatory T cells (nTregs), are responsible for the impaired function of nTregs, resulting in an autoimmune disease known as the immune dysregulation, polyendocrinopathy, enteropathy, X-linked (IPEX) syndrome. The relevance of other peripheral tolerance mechanisms, such as the presence and function of type 1 regulatory T (Tr1) cells, the major adaptive IL-10-producing Treg subset, in patients with IPEX syndrome remains to be clarified. *FOXP3*^mutated^ Tr1-polarized cells, differentiated in vitro from CD4^+^ T cells of four IPEX patients, were enriched in IL-10^+^IL-4^−^IFN-γ^+^ T cells, a cytokine production profile specific for Tr1 cells, and expressed low levels of FOXP3 and high levels of Granzyme-B. IPEX Tr1 cells were hypoproliferative and suppressive, thus indicating that *FOXP3* mutations did not impair their function. Furthermore, we isolated Tr1 cell clones from the peripheral blood of one *FOXP3*^null^ patient, demonstrating that Tr1 cells are present in vivo and they can be expanded in vitro in the absence of WT FOXP3. Overall, our results (i) show that functional Tr1 cells differentiate independently of FOXP3, (ii) confirm that human Tr1 and nTregs are distinct T-cell lineages, and (iii) suggest that under favorable conditions Tr1 cells could exert regulatory functions in IPEX patients.

## Introduction

Peripheral T-cell tolerance can be induced and maintained by a variety of mechanisms, including deletion, induction of T-cell anergy, and differentiation of Tregs. Among the different subsets of T cells with suppressive activity so far described, CD4^+^ Tregs are the most well-defined ones. Naturally occurring CD4^+^CD25^+^ Tregs (nTregs) typically arise in the thymus and their function and maintenance are dependent on high and stable forkhead box p3 (FOXP3) expression, the master gene regulator of nTreg function in both mice and humans 1. We previously demonstrated that nTregs have varying functional impairment in patients carrying mutations in *FOXP3* 2, who develop a life-threatening autoimmune disease known as immune dysregulation, polyendocrinopathy, enteropathy, X-linked (IPEX) syndrome. This disease is mainly characterized by enteropathy, diabetes mellitus, thyroiditis, eczema, and elevated IgE serum levels 3, 4.

In addition to thymus-derived nTregs, adaptive CD4^+^ Tregs can differentiate in the periphery from naïve precursors under various tolerogenic conditions. Among these, the type 1 regulatory (Tr1) cell subset is typically defined based on the cytokine production profile (IL-10^high^, TGF-β^+^, IL-5^+^, IFN-γ^+^, IL-2^low^, and IL-4^−/low^) 5. Accumulating evidence indicates that Tr1 cells play a key role in regulating adaptive immune responses in vivo in both mice and humans, and thus making them potential candidates for use in cell-based therapies for immune-mediated diseases 6, 7.

Lineage independence of Tr1 and nTregs has been investigated using murine transgenic models 8; results from this study indicate the existence of a FOXP3^−^ IL-10-producing regulatory cell subset, thus suggesting that in mice Tr1-like cells do not require FOXP3 for their differentiation and survival. In early studies, in human healthy subjects, we have demonstrated that nTregs and Tr1 cells are independent subsets, showing that Tr1 cells can arise in vitro in the absence of CD4^+^CD25^+^ Tregs 9. Additional studies by us and others have shown that, differently from nTregs, Tr1 cells do not express constitutive CD25 or FOXP3, but they can transiently upregulate both markers upon activation 10–12. More recently, it has been demonstrated that a subset of CD4^+^FOXP3^−^ T cells possesses IL-10-dependent regulatory activity 13. On the other hand, it has been reported that CD4^+^CD25^+^ Tregs may also suppress effector T (Teff) cell responses through the production of IL-10 and TGF-β 14–16 and that human Tr1 cell clones are converted to Th2 cells upon knockdown of FOXP3 17, 18. Thus based on the available data, the lineage distinction of these two Treg subsets is still unclear. Data showing preserved IL-10 production by PBMCs of one IPEX patient anticipate that *FOXP3*^mut^ T cells could be the appropriate model to address this issue 19.

In the present study, we took advantage of the unique opportunity to study patients with IPEX syndrome (i) to unravel the developmental and functional relationship between nTregs and Tr1 cells and (ii) to assess whether Tr1 cells could compensate for nTreg deficiency in IPEX. To these aims, we investigated both in vitro and in vivo development of Tr1 cells in the absence of functional FOXP3. Overall, our results show that Tr1 cell differentiation is independent of FOXP3 and that suppressor IL-10-producing T cells can arise in vivo in the absence of WT FOXP3.

## Results

### *FOXP3*^mutated (mut)^ naïve CD4^+^ T cells can differentiate in vitro into Tr1 cells

**Figure 1:**
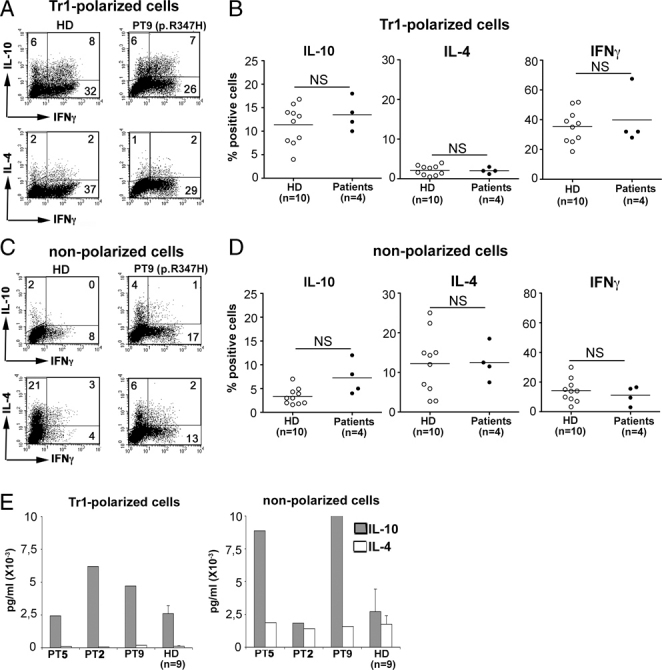
Cytokine production profile of *FOXP3*^mut^ CD4^+^ naïve T cells differentiated under Tr1-polarizing conditions. Cytokine production by (A, B) Tr1-polarized and (C, D) nonpolarized T cells was determined by intracellular staining after stimulation with immobilized anti-CD3 mAb and TPA. Dot plots of (A) Tr1-polarized and (C) nonpolarized cells from one representative HD and one patient are shown. Numbers in the plots indicate the percentage of positive cells in the gate. Gates were set based on isotype controls. The full gating strategy is shown in Supporting Information Fig. 2A. The graphs summarize the percentage of IL10^+^ (left panels), IL4^+^ (middle panels), and IFN-γ^+^ (right panels) cells detected in (B) Tr1-polarized and (D) nonpolarized cell cultures from patients (*n*=4) and HDs (*n*=10). Horizontal line indicates the mean. (E) IL-10 (grey bars) and IL-4 (white bars) released in cell-culture supernatant by Tr1-polarized (left panel) and nonpolarized (right panel) T cells upon anti-CD3 and anti-CD28 mAbs stimulation. Mean±SE of *n*=9 HDs is plotted in the graphs. Statistical analysis was performed with the nonparametric Mann–Whitney test and *p*-values were >0.05 for all plots.

We activated CD4^+^CD45RO^−^ T cells isolated from four IPEX patients (Pt) (Pt2, 5, 9, and 12, all carrying different *FOXP3* mutations) by anti-CD3 cross-linked to CD32^+^ L cells, as artificial APCs, in the presence of IL-10 and IFN-α, as described previously 20. Activation of healthy donor (HD) CD4^+^ naïve T cells under these culture conditions resulted in the differentiation of a distinct population of T cells with a Tr1-like cytokine production profile, as shown by intracellular staining (Fig. 1). In these culture conditions, a subset of T cells produced IL-10 (% IL-10^+^ T cells: mean±SE: 11±1, *n*=10), whereas a markedly lower proportion of cells produced IL-4 (% IL-4^+^ T cells: mean±SE: 2±0.4, *n*=10), in comparison to cells cultured in nonpolarizing conditions (Th0) (% IL-10^+^ T cells: mean±SE: 3±0.5; % IL-4^+^ T cells: mean±SE: 11±2, *n*=10) (Fig. 1A and B). Similar to HD Tr1-polarized T cells, CD4^+^ naïve T cells from IPEX patients cultured in the presence of IL-10 and IFN-α differentiated in a population enriched of IL-10-producing T cells (% IL-10^+^ T cells: mean±SE: 13±2, *n*=4), with low proportion of IL-4-producing cells (% IL-4^+^ T cells: mean±SE: 2±0.5, *n*=4), as compared with control Th0 T-cell cultures (% IL-10^+^ T cells: mean±SE: 7±2, *n*=4; % IL-4^+^ T cells: mean±SE: 12±2, *n*=4), in which a higher fraction of cells produced IL-4 (Fig. 1C and D). Both HD- and patient-derived Tr1-polarized cells contained a substantial proportion of IFN-γ-producing cells (HD % IFN-γ^+^ T cells: mean±SE: 36±3, *n*=10; Pt % IFN-γ^+^ T cells: mean±SE: 40±10, *n*=4) part of which also produced IL-10 (HD % IL-10^+^IFN-γ^+^ T cells: mean±SE: 7±1, *n*=10; Patients % IL-10^+^IFN-γ^+^ T cells: mean±SE: 9±3, *n*=4) (Fig. 1A and B).

High IL-10 and low IL-4 production by Tr1-polarized cells in both patients (*n*=3) and HDs (IL-10 pg/mL: mean±SE: 2600±610; IL-4 pg/mL: mean±SE: 120±40, *n*=9) were also detected in cell-culture supernatants (Fig. 1E). Patients' and HD' nonpolarized cells produced variable amounts of IL-10 (HD IL-10 pg/mL: mean±SE: 2710±1720, *n*=9; Patients: Pt5: 8700, Pt2: 1830, Pt9: 10 000 pg/mL), but concomitant high IL-4 production was also detected (HD IL-4 pg/mL: mean±SE: 1750±640, *n*=9; Patients: Pt5: 1880, Pt2: 1420, Pt9: 1580 pg/mL), resulting in an overall different cytokine production profile, with an IL-10/IL-4 ratio lower than in Tr1-polarized cells (HD IL-10/IL-4 ratio Tr1 versus Th0: 22 versus 2; Patients IL-10/IL-4 ratio Tr1 versus Th0: 36 versus 4).

These results indicate that a T-cell population with a Tr1-like cytokine production profile can differentiate in vitro from naïve CD4^+^ T cells carrying mutations in the *FOXP3* gene, suggesting that FOXP3 is not necessary for in vitro differentiation of Tr1 cells.

### Tr1-polarized T cells from both HD and IPEX patients express low FOXP3 and CD25 and high Granzyme B

**Figure 2:**
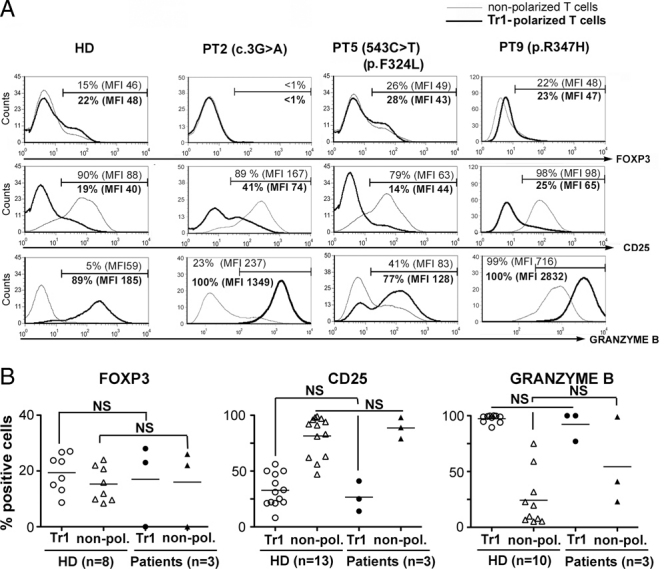
Phenotypic characterization of *FOXP3*^mut^ CD4^+^ naïve T cells differentiated in Tr1-polarizing conditions. (A) Cell-surface CD25 (middle panels) and intracellular FOXP3 (upper panels) and Granzyme B (lower panels) expression in nonpolarized (thin line) or Tr1-polarized (thick line) cells differentiated from naïve CD4^+^ T cells of patients and one representative HD are shown. Numbers in the plots indicate the percentage and MFI of positive cells. The full gating strategy is shown in Supporting Information Fig. 2B. (B) The graphs summarize the data obtained from T cells of HDs (white symbols) and patients (black symbols) differentiated in Tr1-polarized (circles) or nonpolarizing (triangles) conditions at the end of 2-wk culture without further reactivation. Horizontal lines indicate the mean. Statistical analysis was performed with the nonparametric Mann–Whitney test. NS, not significant.

To assess whether Tr1-polarized cell cultures were enriched of FOXP3-expressing cells, FOXP3 expression was detected by flow cytometric analysis. Similar to nonpolarized culture conditions, differentiation in the presence of IL-10 and IFN-α did not induce strong upregulation of FOXP3 expression in HD T cells (Fig. 2A). Only a small fraction of Tr1-polarized T cells expressed FOXP3, compatibly with repetitive activation and culture in the presence of IL-2 and IL-15 21 (%FOXP3^+^ T cells: range: 9–27, mean±SE: 19±2, *n*=8). Tr1-polarized T-cell cultures generated from naïve T cells of Pt5 and Pt9 (who carry *FOXP3* mutations which do not abrogate protein expression, as reported previously 22, 23), displayed levels of FOXP3 expression comparable to both autologous nonpolarized controls and to HD Tr1 cells (Fig. 2A, upper panels and Fig. 2B). In T-cell cultures derived from naïve T cells of Pt2, FOXP3 expression was not detectable in both Tr1-polarized and control nonpolarized T cells (Fig. 2A), due to the presence of a *FOXP3*^null^ mutation in the T cells of this patient, as confirmed by *FOXP3*-sequence analysis of genomic DNA of the polarized cell cultures (data not shown).

Further phenotypic characterization of Tr1-polarized T-cell cultures showed reduced CD25 expression, as compared with nonpolarized controls in both HD and patient-derived cell cultures (Fig. 2A, middle panels and Fig. 2B). Similar to HD Tr1-polarized cells, *FOXP3*^mut^ Tr1 cells expressed higher levels of Granzyme B than their nonpolarized counterparts (Fig. 2A, lower panels and Fig. 2B), whereas the expression of Granzyme A was similar in all culture conditions (data not shown). Therefore, Granzyme B expression, which characterizes Tr1 cells 24, 25 and is typically induced by IL-10 26, appears to be independent of FOXP3 since both HD and patients' Tr1-cell populations upregulate this marker.

Expressions of the cell-surface molecules IL-15R, CD122 (IL-2Rβ chain), and CD132 (IL-2Rγ chain), previously reported to be upregulated on Tr1 cells 27, and of the intracytoplasmic Treg-associated marker cytotoxic T-lymphocyte antigen 4 (CTLA-4) were also tested, but no difference between Tr1-polarizing and nonpolarizing conditions was detected, most likely due to the prolonged culture in the presence of IL-2 and IL-15 (data not shown). Overall, the phenotypic analysis of *FOXP3*^mut^ Tr1-polarized cells confirms that FOXP3 is expressed at low levels in Tr1 cells and demonstrates that *FOXP3*^mut^ Tr1 cells are phenotypically similar to their WT counterpart.

### *FOXP3*^mut^ Tr1-polarized T cells display in vitro suppressive activity

In addition to their cytokine production profile, Tr1 cells are functionally characterized by their intrinsic low proliferative capacity and in vitro suppressive function 10, 27, 28.

**Figure 3:**
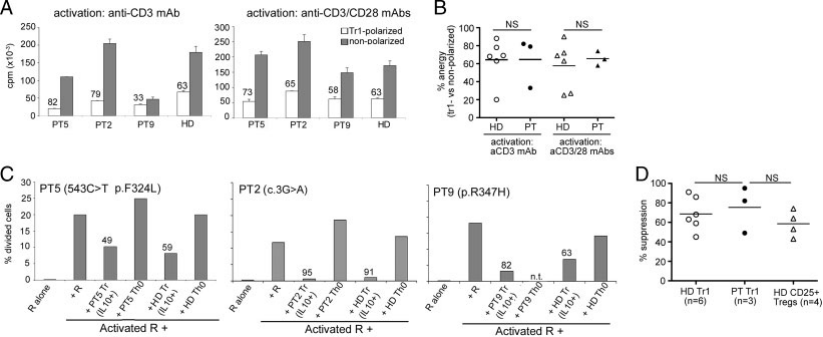
Functional characterization of *FOXP3*^mut^ CD4^+^ naïve T cells differentiated in Tr1-polarizing conditions. (A) Tr1-polarized (white bars) and nonpolarized (grey bars) cells were stimulated with immobilized anti-CD3 mAb alone (left panel) or plus anti-CD28 mAb (right panel). Numbers in the plots indicate the percent anergy calculated versus nonpolarized controls. (B) The graph summarizes the percent anergy calculated versus nonpolarized controls of all HDs (*n*=6) and patients (*n*=3) tested. Horizontal lines indicate the mean. Statistical analysis was performed with the nonparametric Mann–Whitney test, *p*-value was >0.05 for both groups. (C) The capacity of Tr1-polarized cells differentiated from naïve T cells of patients to suppress the proliferation of allogeneic CD4^+^ T cells was evaluated by CFSE dilution assay. Numbers in the plots indicate the percentage inhibition of proliferation. n.t., not tested; R, responder T cells. The full gating strategy is shown in Supporting Information Fig. 3. (D) The graph summarizes the percentage inhibition of proliferation by Tr1-polarized cells from the three patients tested in (C) (black circles) and a pool of HDs (white circles). As a control, the percentage suppression of CD25^+^ Tregs freshly isolated from the peripheral blood of HDs (white triangles) is also shown. Horizontal lines indicate the mean. Differences in the suppressive capacity of patients' Tr1-polarized cells versus that of HDs' Tr1 cells was evaluated with Mann–Whitney test, *p*-value was >0.05. NS, not significant.

Similar to HD, in comparison to nonpolarized control cultures, *FOXP3*^mut^ Tr1-polarized T cells displayed a reduced proliferative response to TCR-mediated activation in the presence or absence of costimulation (Fig. 3A and B) (% reduction of proliferation of HD IL-10+IFN-α polarized versus nonpolarized T cells: anti-CD3 mAb stimulation range: 20–88%; mean±SE: 64±10%; anti-CD3+anti-CD28 mAbs stimulation range: 25–90%, mean±SE: 58±11%; *n*=6).

To establish the functional capacity of in vitro-differentiated *FOXP3*^mut^ Tr1 cells to act as suppressor cells, we tested whether naïve T cells from IPEX patients differentiated in Tr1-polarizing conditions could suppress the proliferation of allogeneic CD4^+^ T cells as efficiently as HD Tr1 cells. As shown in Fig. 3C, addition of control Th0 cells, primed in the absence of polarizing cytokines, had minimal effect on the proliferation of allogeneic CD4^+^ T cells. On the contrary, T cells cultured in the presence of IL-10 and IFN-α strongly suppressed the proliferation of responder T cells. The percentage of inhibition of Tr1-polarized cells from IPEX patients was comparable to that observed in Tr1 cells differentiated from normal donors (at [responder:suppressor] ratio of [1:1], range: 45–91%; mean±SE: 69±7%; *n*=6) and to the suppressive capacity of CD25^+^ Tregs freshly isolated from HDs and tested under the same culture conditions (range: 43–74%; mean±SE: 59±7%; *n*=4) (Fig. 3C and D). The observation that CD4^+^ T cells differentiated with IL-10 and IFN-α display a hypoproliferative response to TCR stimulation independently of FOXP3 expression suggests that their anergic phenotype, as their suppressive activity, is not dependent on FOXP3.

### Functional Tr1-cell clones can be isolated from the peripheral blood of patients with IPEX syndrome

To determine whether Tr1 cells could arise in vivo in IPEX patients, T-cell clones were randomly generated starting from peripheral CD4^+^ T cells of a patient carrying a *FOXP3*^null^ mutation (Pt2, mutation: 3G>A 22, 29). Pt2 received bone marrow transplant (BMT), but donor chimerism decreased rapidly after transplant, remained persistently low, and in the CD4^+^ T-cell compartment was below 10% at the time of T-cell cloning 29.

We selected T-cell clones that upon activation produced IL-10, with concomitant low IL-4, as potential Tr1-cell clones for further characterization of cytokine production, DNA sequence analysis for host/donor origin identification and functional properties.

**Figure 4:**
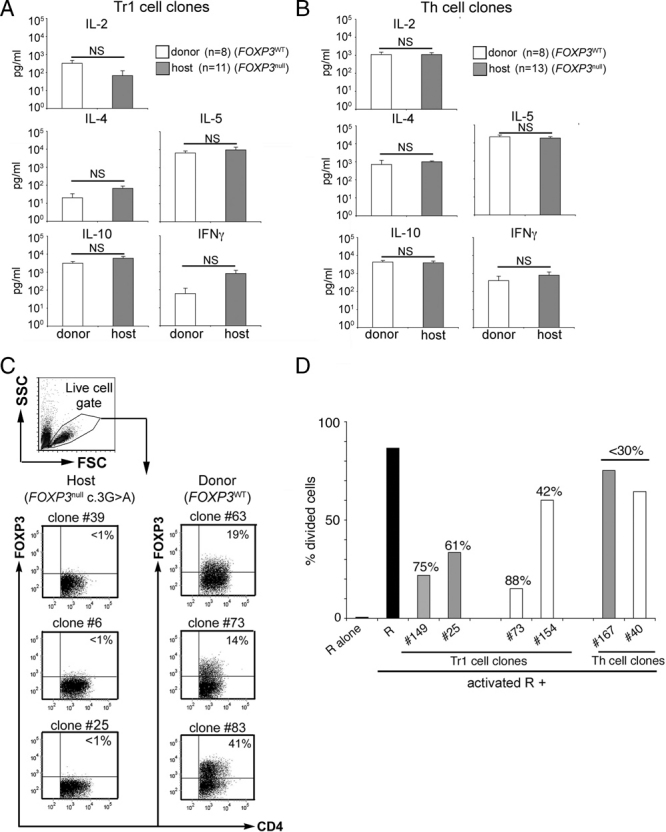
Characterization of ex vivo-isolated *FOXP3*^null^ T-cell clones. Cytokine production profile of Tr1 (A) and Th (B)-cell clones of host (grey bars) and donor (white bars) origin was determined upon anti-CD3 plus anti-CD28 mAbs stimulation. Results are presented as mean±SE. Statistical analysis was performed with the two-tailed unpaired Student's *t*-test, *p*>0.05 for all cytokines. NS: not significant. (C) T-cell clones of host (left panels) and donor (right panels) origin were activated for 48 hours in the presence of anti-CD3/anti-CD28 mAbs and IL-2 (100 U/mL) prior to FOXP3 staining. Representative T-cell clones of host and donor origin are shown. Numbers in the plots indicate the percentage of positive cells in the quadrant. T cells were gated on Live cell gate, and identified based on the physical parameters, as shown. (D) The ability of *FOXP3*^null^ (grey bars) Tr1-cell clones to suppress CD4^+^ allogeneic responder (R) T cells was assessed by CFSE dilution assay. The full FACS gating strategy is shown in Supporting Information Fig. 3. *FOXP3*^WT^ (white bars) Tr1-cell clones of donor origin were tested in parallel. As control, both *FOXP3*^null^ and *FOXP3*^WT^ Th-cell clones were also tested. Numbers in the plots indicate the percentage inhibition of proliferation. Data are representative of two independent experiments.

The presence of *FOXP3*^null^ T-cell clones with a Tr1-like cytokine production profile was confirmed by the analysis of cytokines in supernatants of activated T cells. In this analysis, we classified a T-cell clone as Tr1 when the IL-10/IL-4 ratio was at least 30-fold (high IL-10/IL-4 ratio is a hallmark of Tr1-cell clones 30), with concomitant negative or low IL-2 production. T-cell clones with IL-10/IL-4 ratio over 100 were classified as Tr1-cell clones independently of IL-2 production. The majority of T-cell clones analyzed, either Tr1- or Th-cell clones, were of host origin (Fig. 4A and B), in accordance with low donor chimerism in peripheral blood.

Overall, 60% of the T-cell clones with a Tr1-like cytokine production profile were of host origin (Fig. 4A and Supporting Information Tables 1 and 2) and the amount of cytokines produced was comparable to Tr1-cell clones of donor origin (Fig. 4A), suggesting that the presence of a *FOXP3*^null^ mutation does not hamper in vivo differentiation of Tr1 cells. The complete absence of FOXP3 protein translation was confirmed by FACS analysis of activated T-cell clones of donor and host origin carrying the *FOXP3*^null^ mutation (Fig. 4C).

Similar to HD Tr1-cell clones 10, 28, *FOXP3*^mut^ Tr1-cell clones displayed reduced proliferative capacity in response to TCR-mediated activation, as compared with Th-cell clones of host origin, used as control (mean % reduction: 46 versus *FOXP3*^mut^ Th-cell clones; Tr1 *n*=5; Th *n*=7) (data not shown). Moreover, *FOXP3*^mut^ Tr1-cell clones were able to suppress the proliferative response of allogeneic CD4^+^ T cells as efficiently as *FOXP3*^WT^ Tr1-cell clones, tested in parallel (Fig. 4D). T-cell clones of host origin displaying a low IL-10/IL-4 ratio were used as control Th-cell clones (Fig. 4D). Among the T-cell clones with a Tr1-like cytokine production profile tested for in vitro suppression, two out of three T-cell clones expressing WT FOXP3 significantly reduced the proliferation of allogeneic CD4^+^ T cells (>30% suppression at [CD4+responders:suppressors] ratio of [1:1]). Similar to *FOXP3*^WT^ Tr1-cell clones, two out of five of the *FOXP3*^mut^ Tr1-like cell clones tested (*n*=5) displayed efficient in vitro suppressive activity (Supporting Information Table 3). Furthermore, blockade of IL-10 and TGF-β, by adding to the co-culture anti-IL-10R and anti-TGF-β mAbs, partially reverted the suppressive activity of both *FOXP3*^WT^ and *FOXP3*^null^ Tr1-like cell clones (% reversion of suppressive activity range was 16–41% for *FOXP3*^WT^ Tr1-cell clones, *n*=3 and 13–100% for *FOXP3*^mut^ Tr1-cell clones, *n*=2) (Supporting Information Fig. 1). In addition, the suppressive activity of a FOXP3^+^ Treg clone generated from FACS sorted CD4^+^CD25^bright^ T cells of a HD, used as control, was not reverted in the presence of anti-IL-10R and anti-TGF-β mAbs (Supporting Information Fig. 1).

Overall, these results demonstrate that both *FOXP3*^null^ and *FOXP3*^WT^ Tr1-cell clones showed similar in vitro functional properties and that suppressive activity of the *FOXP3*^null^ Tr1 cells is indeed IL-10- and TGF-β-dependent, as previously demonstrated for WT Tr1 cells 11, 31.

**Figure 5:**
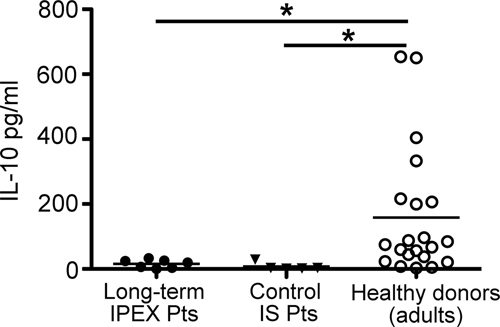
IL-10 production by PBMCs isolated from patients with IPEX syndrome. PBMCs were activated with anti-CD3/CD28 mAbs for 72 h. *n*=7 independent determinations of *n*=4 long-term patients (Pt9, 11, 14, and 20, all under immunosuppressive treatment since at least 4 years) (black circles), of *n*=5 non-IPEX patients (black triangles), treated with multiple immunosuppressive drugs, and of *n*=21 adult HDs (white circles) are shown. Horizontal lines indicate the mean. Statistical analysis was performed with the nonparametric Mann–Whitney test. ^*^p<0.05.

We further investigated the presence of circulating IL-10-producing cells in the peripheral blood of patients with IPEX syndrome by evaluation of IL-10 production by patients' PBMCs upon TCR-mediated stimulation. As shown in Fig. 5, low IL-10 production was detected in long-term IPEX patients, young adults who overcame the initial acute phase of the disease and were under treatment with immunosuppressive drugs (Table 1). Low IL-10 release by peripheral T cells was also detected in a group of non-IPEX patients, without detectable *FOXP3* mutation, but with autoimmune manifestations of unknown origin (most of them displayed enteritis) kept under control by multiple immunosuppressive treatments (Fig. 5). These patients served as control group to assess the impact of IS on in vitro IL-10 production upon TCR-mediated stimulation. However, phenotypic analysis of patients' CD4^+^CD25^−^CD127^−^ T cells, a T-cell population recently described to include a fraction of memory IL-10-producing cells with regulatory activity 13, revealed frequencies similar to healthy controls (data not shown). Overall, these data suggest that, although present and normally differentiating, Tr1 cells in IPEX patients are not as efficient as those in healthy control.

**Table 1:** Summary of the clinical, immunological, and molecular characterization of IPEX patientsa)

Pt	*FOXP3* mutation	Age (years)	Age at onset	Enteropathy	Endocrinopathy	Skin disease	Other	Therapy
2b)	c.3G>A	5	Neonatal	Severe diarrhea w VA	IDDM hypothyroidism	Severe eczema	Lymphadenopathy hepatosplenomegaly	MUD
5b)	p.F324L; 543C>T	7	Neonatal	Severe diarrhea w eosinophilic infiltration w/o VA	–	Mild eczema	Allergic asthma	–
9b)	p.R347H	14	Neonatal	Severe diarrhea w VA	IDDM	Mild eczema	Hepatitis/thrombocytopenia/Coombs neg anemia /food allergy/FT	IS (low steroids)
11b)	p.A384T	15	Neonatal	Severe diarrhea w eosinophilic infiltration w/o VA	IDDM thyroiditis	Severe eczema/alopecia	AEA/interstitial pneumonia/FT	IS (multiple drugs)
12b)	p.F373A	7	Neonatal	Severe diarrhea w VA	IDDM	Eczema	–	HLAid
14b)	p.L242P	14	4 mo	Severe diarrhea w VA	–	Mild eczema	Sepsis/nephropathy	IS (multiple drugs)
20c)	c.816+2del	27	5 mo	Severe diarrhea w VA	–	Eczema	Thrombocytopenia/arthritis/sepsis/bronchitis	IS (multiple drugs)

a) mo, months; VA, villous atrophy; w, with; w/o, without; IDDM, insulin-dependent diabetes mellitus; neg, negative; FT, failure to thrive; AEA, autoimmune hemolytic anemia; MUD, matched unrelated donor; IS, immunosuppression; and HLAid, HLA identical.

b) *FOXP3* mutations and clinical history were previously reported in 19.

c) Pt20 carries a previously undescribed mutation at the 5′ donor splice site of intron 7 (NM_014009.3).

## Discussion

In the present study, we show that suppressor Tr1 cells can differentiate in vitro from naïve CD4^+^ T cells isolated from IPEX patients, carrying different mutations in the *FOXP3* gene. Furthermore, we demonstrate that Tr1-cell clones are present in the peripheral blood of a patient carrying a *FOXP3*^null^ mutation. These results indicate that FOXP3 is not required for both in vitro and in vivo differentiation of Tr1 cells. Tr1 cells obtained in vitro or isolated ex vivo from IPEX patients display all the features commonly adopted to define Tr1 cells 5. In vitro-differentiated FOXP3^mut^ Tr1 cells are hypoproliferative, produce high levels of IL-10, with concomitant low IL-4 and IL-2, express high levels of Granzyme B and low CD25 and, most importantly, display in vitro suppressive activity. The above-mentioned properties are also shared by *FOXP3*^mut^ Tr1-cell clones induced in vivo. Nonetheless, clinical evidence demonstrates that the lack of nTregs in infants with IPEX syndrome is not fully compensated by the induction of functional Tr1 cells.

Similar to *FOXP3*^WT^ Tr1 cells, *FOXP3*^mut^ Tr1 cells or Tr1-cell clones consistently show high IL-10 production and in vitro suppressive capacity. Furthermore, *FOXP3*^mut^ cells induced in vitro 11, 12 or isolated ex vivo show low FOXP3 expression, whose intensity may vary based on the state of activation of the cells at the time of analysis, as reported previously. These findings support the notion that FOXP3 is not important for the maintenance of Tr1-cell features and function 11, 12. Indeed, IL-10-producing T cells have been previously obtained from one IPEX patient after short-term in vitro stimulation 19. Our results not only extend these findings to patients with different *FOXP3* mutations, thus demonstrating that Tr1-cell fate is independent from FOXP3, but also provide evidence that *FOXP3*^mut^ Tr1 cells can differentiate in vivo and display features and function similar to *FOXP3*^WT^ Tr1 cells.

Although lineage relationship between Tr1 cells and nTregs has not been fully unraveled, using transgenic mice with a dual-reporter system of the genes encoding IL-10 and Foxp3, it has been previously demonstrated that three distinct Treg subsets exist: Foxp3^+^IL-10^+^, Foxp3^+^IL-10^−^, and Foxp3^−^IL-10^+^ (defined as Tr1-like) Tregs and their relative frequency varies depending on the tissue compartment 8. These results indicate that in the mouse, induction of IL-10 expression is initiated extrathymically regardless of Foxp3 expression 8. Similarly, human Tr1-cell differentiation in vitro does not require CD4^+^CD25^+^ Tregs 11. Here, we provide definitive evidence that the expression of FOXP3 is not necessary for Tr1-cell differentiation or function, thus indicating that nTregs and Tr1 cells in humans are two distinct subsets of cells with regulatory activity.

Importantly, the isolation of T-cell clones with a Tr1-like cytokine production profile from the peripheral blood of a transplanted patient with a *FOXP3*^null^ mutation indicates that functional Tr1 cells can arise in vivo regardless of FOXP3 expression. In apparent contrast to these evidences, it has been recently reported by Veldman et al. that human Tr1-cell clones specific for desmoglein3 (Dsg3) constitutively express FOXP3 and knockdown of FOXP3 expression results in loss of Tr1 phenotype and function 17, 18. Notably, in their studies, FOXP3 expression in Tr1-cell clones was measured exclusively by qPCR analysis 17, 18. Tr1 cells, as well as peripheral CD4^+^CD25^+^ T cells, displayed only a four-fold increase in FOXP3 expression as compared with purified CD4^+^CD25^−^ Teff cells, corresponding to a low level of FOXP3 expression, comparable to that of activated Teff cells, as already reported by us and others 11, 32, 33. In addition, the observed lack of suppressive activity by Tr1 cell clones upon FOXP3 knockdown may be due to subsequent reversion of the hypoproliferative state of T cells, as already demonstrated for nTregs 34, rather than to a specific function for FOXP3 in the suppressor activity of Tr1 cells 18.

Overall, it is relevant that we isolated Tr1 cells carrying a *FOXP3*^null^ mutation several years after transplant, in the absence of immunosuppression, in a chimeric environment. Indeed, in other mixed chimeric patients, who underwent HSCT for diseases other than IPEX, Tr1 cells are key players in the maintenance of persistent mixed chimerism 27, 30. In this patient, nTregs are entirely donor-derived, and therefore fully *FOXP3*^WT^ 29, whereas Tr1 cells are of both host (*FOXP3*^null^) and donor (*FOXP3*^WT^) origin and it is likely that they are operational in vivo in maintaining tolerance. Although based on the number of T-cell clones obtained upon in vitro culture we cannot evaluate the peripheral frequency of Tr1 cells of host and donor origin, our data suggest that in vivo *FOXP3*^WT^ Tr1 cells, differently from nTregs, do not display preferential growth, as compared with their *FOXP3*^null^ counterpart.

However, in IPEX patients, the rapid development of the wasting autoimmune disease after birth indicates that, although inducible, Tr1 cells are not sufficient by themselves to control autoimmunity, especially not at the onset of the disease. We could speculate that the strong inflammatory environment that characterizes IPEX syndrome at the onset 35, 36 interferes with Tr1-cell induction, which requires persistent antigen exposure in tolerogenic environmental conditions 37, 38. Alternatively, it cannot be excluded that WT FOXP3 expression in T cells is required for interaction with APCs in order to induce a tolerogenic environment suitable for Tr1-cell induction in vivo.

In addition, in those patients who have overcome the initial acute phase and have become stable with a milder form of the disease controlled by pharmacological immunosuppression, we observed low IL-10-production. This suggests that at this stage of the disease Tr1-cell function may be prevented by the presence of immunosuppressants. Therefore, therapeutic strategies aimed at dampening inflammation without interfering with the development and function of Tr1 cells 39 should be preferred to strong immunosuppression. The beneficial effect of rapamycin, reported in several IPEX patients 40, 41, could potentially be ascribed to a similar phenomenon.

In conclusion, our results demonstrate that despite mutations in *FOXP3*, fully functional IL-10-producing Tr1 cells can differentiate, but it remains to be defined to which extent the development of a functional Tr1-cell pool in vivo would allow the control of the acute phase and the progression toward a milder form of the disease. Further investigation is needed to address this issue and define the suitable environmental conditions favoring the development of an efficient Tr1-cell population, not only in IPEX syndrome but also in other pathologies with immune dysregulation.

## Materials and methods

### Patients and cell purification

The clinical and molecular details of the patients (pt) included in the study are summarized in Table 1. Peripheral blood was obtained upon informed consent in accordance with the internal ethical committee of San Raffaele Scientific Institute approval (protocol TIGET-02). Pt12 was studied at the onset of the disease, whereas samples from Pt2, 5, 9, 11, 14, and 20 were obtained several years after onset. Pt2 underwent bone marrow transplant; his cells were studied 4 years after transplant when the donor chimerism was stably below 10% in CD4^+^ T-cell compartment 29. Pt9, 11, 14, and 20 were under immunosuppression at the time of this study. All patients except Pt20 were reported previously 22 (Table 1). In Fig. 5, control patients are subjects with multiple autoimmune manifestations (which include enteropathy, endocrinopathies, and skin diseases) (*n*=5) who were investigated for the presence of mutations in *FOXP3*, but were found to be negative. All patients were under immunosuppressive treatment at the time of determination. PBMCs were purified over Ficoll-Hypaque gradients. CD4^+^ and CD45RO^−^ T cells were isolated by negative selection (Miltenyi Biotec) and were over 90% pure.

### T-cell culture

In vitro Tr1-cell differentiation was performed in the presence of IL-10 and IFN-α, as described previously 20. As a control, T cells were cultured in the absence of polarizing cytokines (hereafter, indicated as nonpolarized or Th0 cultures). Prior to suppression assays, the IL-10-producing fraction of Tr1-polarized cells was enriched with the IL-10-producing-cell isolation kit (Miltenyi Biotec), following the manufacturer's instructions. T-cell clones were obtained from CD4^+^ cells by limiting dilution, as described previously 27 and were expanded with IL-2 40 U/mL and IL-15 10 ng/mL (R&D Systems). All cultures were performed in X-Vivo15 (Bio-whittaker), 5% human serum (Bio-whittaker) plus *penicillin/streptomycin* (Invitrogen).

### T-cell proliferation and suppression assays

In order to evaluate the proliferative response of cultured T cells, 0.5–1×10^5^ cells/well were activated with plate-bound anti-CD3 mAb 1–10 μg/mL (OKT3; Janssen-Cilag), alone or plus soluble anti-CD28 mAb 1 μg/mL (BD Pharmingen). After 72 h, cells were pulsed for 16 h with 1 μCi/well of [^3^H]-thymidine (Amersham Biosciences). To evaluate the suppressive activity, allogeneic CD4^+^ T cells were stained with CFSE (Molecular Probes) and activated with plate-bound anti-CD3 10 μg/mL (Janssen-Cilag) plus soluble anti-CD28 1 μg/mL mAbs (BD Pharmingen) (assay on Pt5, Fig. 2C, panel 1) or with irradiated allogeneic APC plus anti-CD3 mAb 1 μg/mL (Janssen-Cilag) (assays on Pt2, 9; Fig. 2C, panels 2–3). Suppressor cells were added at a ratio of 1:1. To evaluate the suppressive activity of Tr1-cell clones, allogeneic PBMCs were used as responders (*R*) and activated with plate-bound anti-CD3 10 μg/mL (Janssen-Cilag) and soluble anti-CD28 1 μg/mL mAbs (BD Pharmingen). T-cell clones were added either at a ratio of 1:1 or at a ratio of 1:0.5 (CD4^+^ T cell:T-cell clone). To revert the suppressive capacity of Tr1-cell clones, anti-IL-10RB (clone 3F9) 30 μg/mL (BD Pharmingen) and anti-TGF-β(1,2,3) (clone 1D11) 50 μg/mL (R&D Systems) mAbs were added to the coculture. As a negative control, isotype IgGs were added. The percentage of divided *R* was calculated by gating on CD4^+^ cells, as described elsewhere 42. Tr1 cells inhibiting responder cell proliferation less than 30% at [1:1] and 15% at [1:0.5] [R:Tr] ratio were not considered suppressive.

### Cytokine production

A total of 10^6^ T cells/mL were activated with anti-CD3 10 μg/mL (Janssen-Cilag) plus soluble anti-CD28 1 μg/mL mAbs (BD Pharmingen). Supernatants were collected after 24 h for IL-2 detection and 48 h for all other cytokines. The presence of cytokines in culture supernatants from T-cell clones was evaluated by ELISA as described elsewhere 43. IL-10 production by patients' PBMC was evaluated in cell-culture supernatants after 72 h of activation and analyzed with flow-based Bio-Plex suspension array system (Biorad).

### FACS analysis

Anti-CD4, -CD25, and -CD45RO were all from BD Pharmingen. Intracellular staining was used for the detection of Granzyme B (Caltag) and FOXP3 (clone 259D, Biolegend), following the manufacturers' instructions. Intracellular cytokines were detected by flow cytometry using anti-IL-10, IL-4, and IFN-γ mAbs (all from BD Pharmingen), as described by Sornasse et al. 44. Samples were analyzed with FCS Express Pro Software version 3 (De Novo Software).

### Statistical analysis

Results are presented as mean±SE or SD, as indicated in the text, and analyzed with nonparametric Mann–Whitney test or with two-tailed unpaired Student's *t*-test, as indicated in the figure legends. *P*<0.05 was considered significant.

## Abbreviations

FOXP3: forkhead box p3
HD: healthy donors
IPEX: immune dysregulation, polyendocrinopathy, enteropathy X linked
Teff: effector T cell
Tr1: type 1 regulatory T cell
