# Supplementary material for: Functional type 1 regulatory T cells develop regardless of FOXP3 mutations in patients with IPEX syndrome
Source: Eur J Immunol. 2011 Jan 14;41(4):1120–31. doi: 10.1002/eji.201040909 (PMC3107421; doi:10.1002/eji.201040909)
Supplement: Supplementary file 1 [file eji0041-1120-SD1.pdf]

Supplemental Table 1. Cytokine production profile of Tr1 cell clones of donor origin.

Clone	IL-2 pg/ml	IL-4 pg/ml	IL-5 ng/ml	IL-10 ng/ml	IFNγ pg/ml	IL-10/IL-4 ratio
50	665	0	13,3	2,7	0	>100
73	775	0	0	0,5	0	>100
76	1005	0	16,6	2,6	0	>100
80	75	60	10,0	2,0	0	34
103	0	0	0	0,6	0	>100
154	10	0	5,6	4,0	0	>100
184	0	0	0	6,2	425	>100
262	30	100	NT	6,5	NT	65
Mean ± SE	320±149	20 ± 14	6,5 ± 2,0	3,1 ± 0,8	61 ± 61	

NT: not tested

SE: standard error

activation: anti-CD3 (10µg/ml) plus anti-CD28 (1 µg/ml) mAbs
