# Supplementary material for: Functional type 1 regulatory T cells develop regardless of FOXP3 mutations in patients with IPEX syndrome
Source: Eur J Immunol. 2011 Jan 14;41(4):1120–31. doi: 10.1002/eji.201040909 (PMC3107421; doi:10.1002/eji.201040909)
Supplement: Supplementary file 2 [file eji0041-1120-SD2.pdf]

Supplemental Table 2. Cytokine production profile of Tr1 cell clones of host origin.

Clone	IL-2 pg/ml	IL-4 pg/ml	IL-5 ng/ml	IL-10 ng/ml	IFNγ ng/ml	IL-10/IL-4 ratio
6	0	45	3,1	3,3	0,5	78
15	0	0	7,6	0,5	0,5	>100
18	0	0	24,8	3,0	2,6	>100
25	645	80	2,8	14,9	1,2	>100
39	60	0	0,4	1,6	0	>100
138	0	240	8,8	11,2	2,6	47
149	0	0	0	1,4	0,03	>100
183	0	0	3,7	0,5	0	>100
198	25	125	35,8	11,4	0,2	93
289	0	125	NT	8,5	NT	68
292	0	125	NT	8,1	NT	65
Mean ± SE	66 ± 58	67 ± 24	9,7 ± 4,1	5,9 ± 1,5	0,8 ± 0,4	

NT: not tested

SE: standard error

activation: anti-CD3 (10µg/ml) plus anti-CD28 (1 µg/ml) mAbs
