# Supplementary material for: Functional type 1 regulatory T cells develop regardless of FOXP3 mutations in patients with IPEX syndrome
Source: Eur J Immunol. 2011 Jan 14;41(4):1120–31. doi: 10.1002/eji.201040909 (PMC3107421; doi:10.1002/eji.201040909)
Supplement: Supplementary file 3 [file eji0041-1120-SD3.pdf]

Supplemental Table 3. Suppressive activity of Tr1 cell clones of host and donor origin.

Tr1 clone	origin	% suppression (mean ± SE[a],)	*FOXP3*[c]
73	Donor	82 ± 6 (n=2)[b]	WT[d]
103	Donor	48 ± 13 (n=3)	WT
154	Donor	35 ± 8 (n=3)	WT
6	Host	18 ± 6 (n=2)	Mut[e]
18	Host	11 ± 3 (n=2)	Mut
25	Host	59 ± 17 (n=3)	Mut
39	Host	10 ± 3 (n=2)	Mut
149	Host	52 ± 15 (n=3)	Mut

a) SE: standard error; b) the suppressive activity of Tr1 cell clones was evaluated in *n* independent experiments, as indicated; c) presence or absence of the mutation was evaluated by DNA sequence analysis of the *FOXP3* gene; d) WT: wild type; e) Mut: mutated.
