# Supplementary figures and images for: Functional type 1 regulatory T cells develop regardless of FOXP3 mutations in patients with IPEX syndrome
Source: Eur J Immunol. 2011 Jan 14;41(4):1120–31. doi: 10.1002/eji.201040909 (PMC3107421; doi:10.1002/eji.201040909)

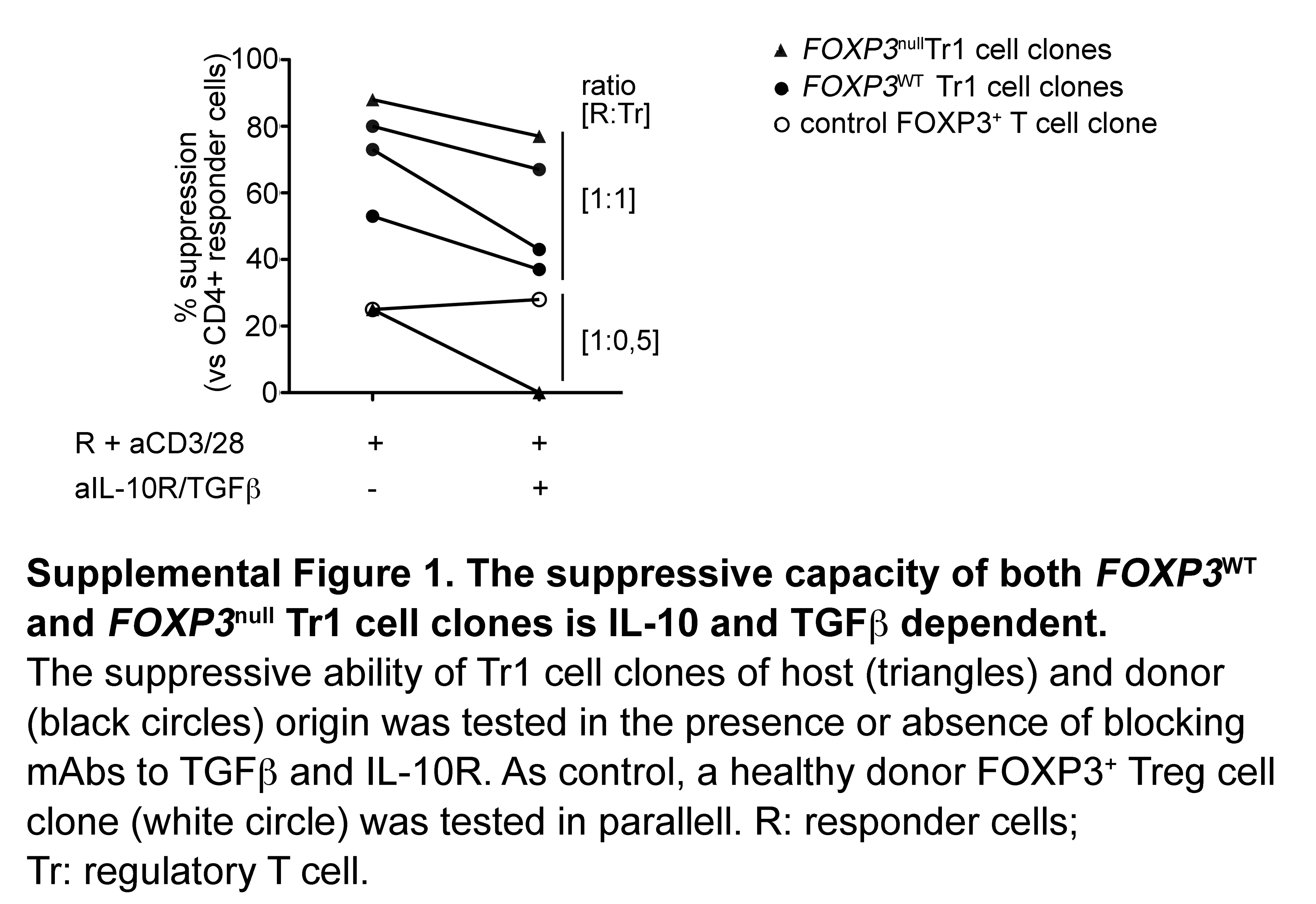

Supplement: Supplementary file 4 [file eji0041-1120-SD4.tif]

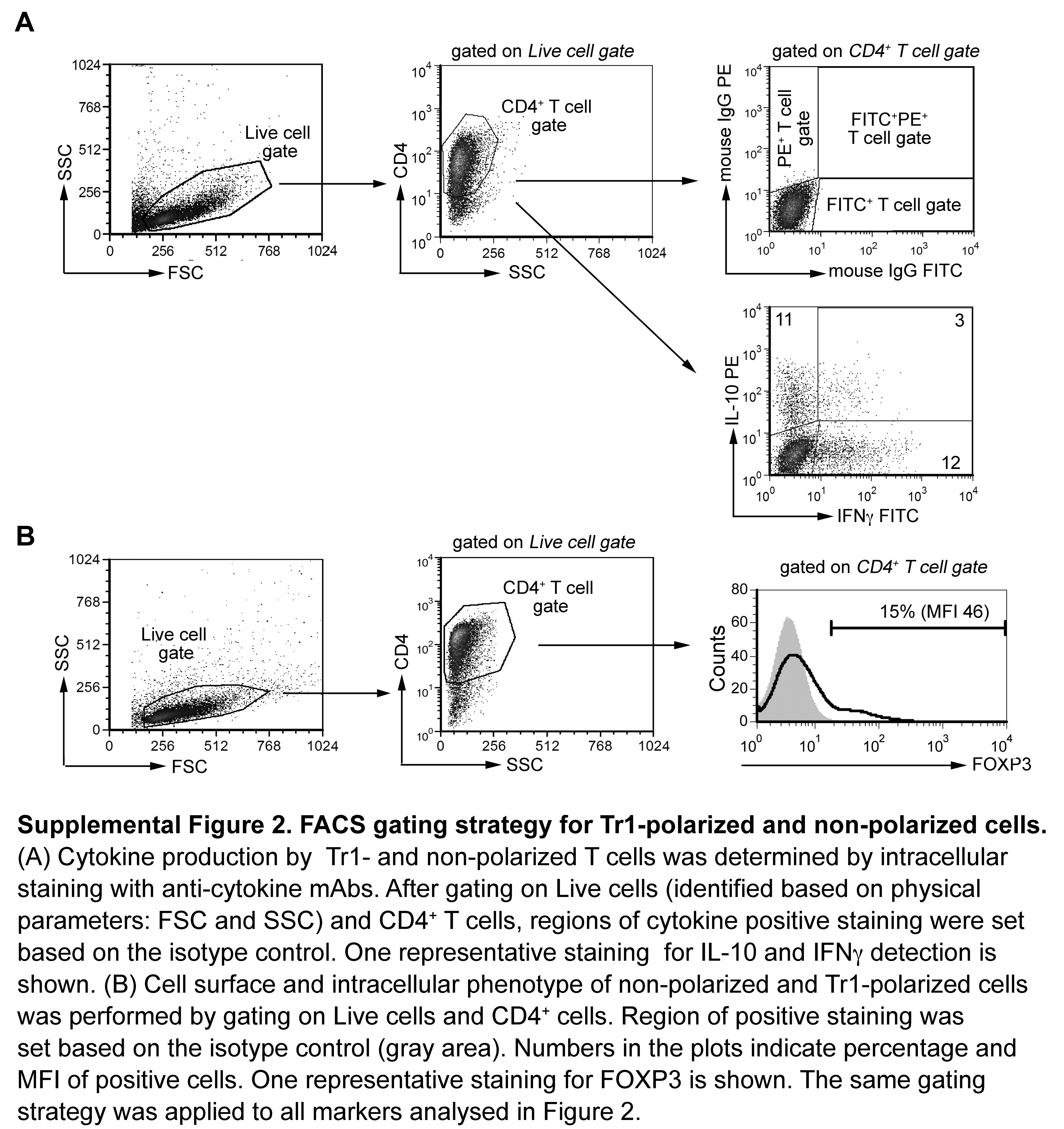

Supplement: Supplementary file 5 [file eji0041-1120-SD5.tif]

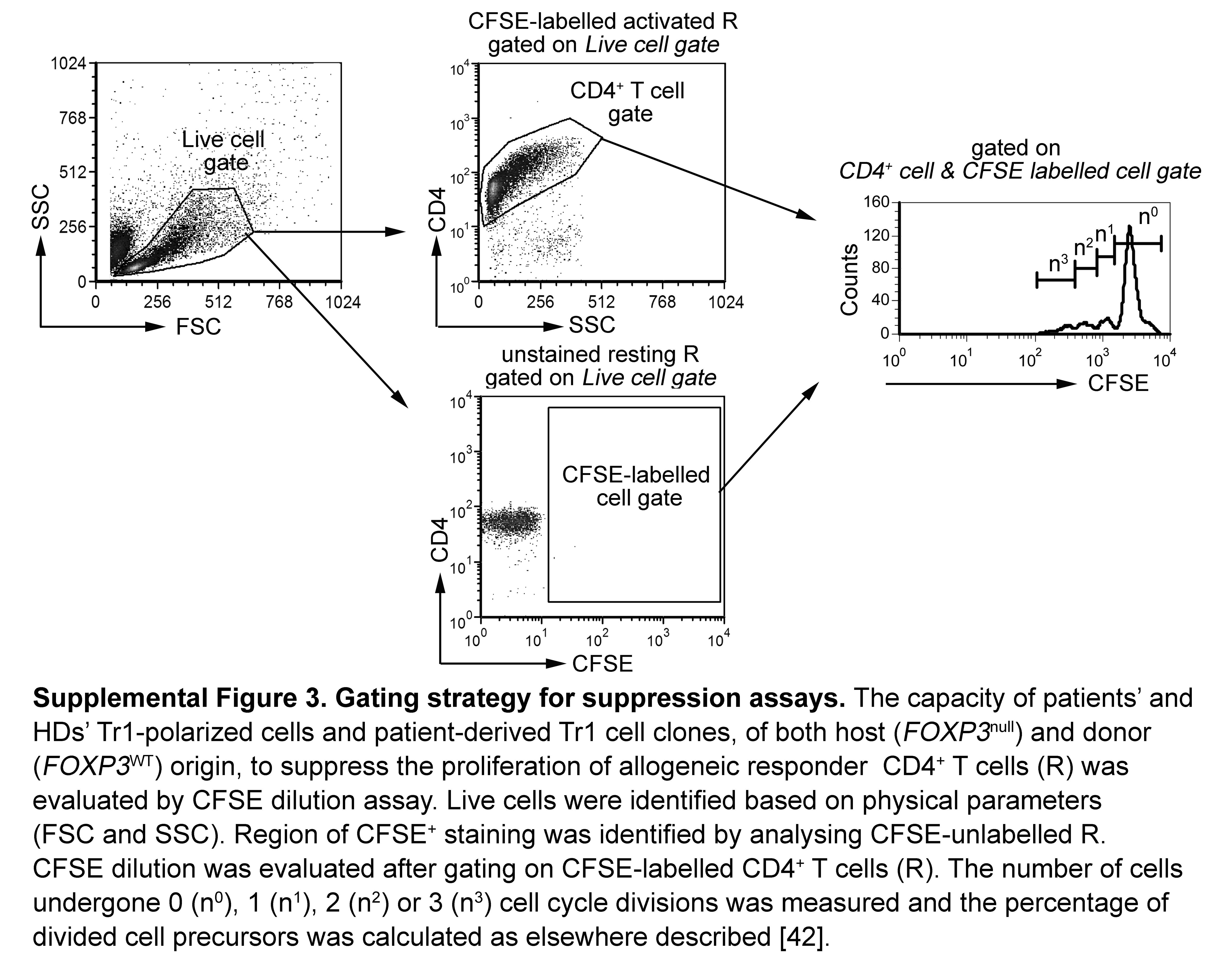

Supplement: Supplementary file 6 [file eji0041-1120-SD6.tif]
