# Supplementary material for: Functional type 1 regulatory T cells develop regardless of FOXP3 mutations in patients with IPEX syndrome
Source: Eur J Immunol. 2011 Jan 14;41(4):1120–31. doi: 10.1002/eji.201040909 (PMC3107421; doi:10.1002/eji.201040909)
Supplement: Supplementary file 7 [file eji0041-1120-SD7.pdf]

European Journal of Immunology

Supporting Information

for

DOI 10.1002/eji.201040909

Functional type 1 regulatory T cells develop regardless of *FOXP3* mutations in patients with IPEX syndrome

Laura Passerini, Sara Di Nunzio, Silvia Gregori, Eleonora Gambineri, Massimiliano Cecconi, Markus G. Seidel, Giantonio Cazzola, Lucia Perroni, Alberto Tommasini, Silvia Vignola, Luisa Guidi, Maria G. Roncarolo and Rosa Bacchetta

© 2011 WILEY-VCH Verlag GmbH & Co. KGaA, Weinheim
